# Health-promoting behavior to enhance perceived meaning and control of life in chronic disease patients with role limitations and depressive symptoms: a network approach

**DOI:** 10.1038/s41598-023-31867-3

**Published:** 2023-03-24

**Authors:** Je-Yeon Yun, Young Ho Yun

**Affiliations:** 1 Seoul National University Hospital, Seoul, Republic of Korea; 2 Yeongeon Student Support Center, Seoul National University College of Medicine, Seoul, Republic of Korea; 3 Department of Biomedical Science, Seoul National University College of Medicine, Seoul, Republic of Korea; 4 Department of Family Medicine, Seoul National University Hospital, 101 Daehak-Ro, Jongno-Gu, Seoul, 03080 Republic of Korea

**Keywords:** Neuroscience, Psychology, Diseases, Health care

## Abstract

The association between health-related role limitations in the mental and physical subdomains and clinical status (i.e., chronic disease and comorbid depressive symptoms) is mediated by health-promoting behaviors. To enhance health-promoting behaviors in adults with chronic disease, it is necessary to identify item-level associations among targets of health-related monitoring and management. Therefore, the current study used a network approach to examine associations among health-related role limitations, depressive symptoms, existential well-being, socioeconomic position, and health-promoting behavior in adults with chronic disease. A total of 535 adults (mean ± SD age = 62.9 ± 11.9 years; males, n = 231, females, n = 304) who were regularly visiting an outpatient clinic for chronic disease treatment participated in this cross-sectional study. Data on participant demographics, chronic disease diagnoses, socioeconomic status, health-related role limitations (12-item short form survey scores), depressive symptoms (patient health questionnaire-9 scores), existential well-being (scores for four items of the McGill quality of life questionnaire-Revised), and health-promoting behavior (Healthy Habits Questionnaire scores) were acquired. “Undirected regularized partial correlations” and “directional joint probability distributions” among these variables were calculated using a mixed graphical model (MGM) and directed acyclic graph (DAG). In the MGM, the most influential nodes were emotional well-being, feelings of failure, and health-related limitations affecting usual role and physical activities. According to both the MGM and DAG, the relationship between emotional well-being and feelings of failure mediated the relationships of health-related role limitations with concentration difficulty and suicidal ideation. A positive mindset was dependent on the probability distributions of suicidal ideation, controllability of life, and positive self-image. Both the meaning of life and a positive mindset had direct associations with proactive living. Specifically, proactive living was associated with a balanced diet, regular exercise, volunteering in the community, and nurturing intimacy in social interactions. The meaning and controllability of life in individuals with chronic diseases could mediate the relationships of health-promoting behavior with health-related limitations related to usual role activities, physical activities, and depressive symptoms. Thus, interventions targeting health-promoting behaviors should aim to enhance the meaning and controllability of life (as it pertains to limitations in usual role and physical activities), as well as promote proactive screening and timely psychiatric treatment of depressive symptoms including feelings of failure, concentration difficulties, and suicidal ideation.

## Introduction

### Importance of health-promoting behavior in the treatment of chronic disease patients

Chronic disease can be defined as a medical condition lasting ≥ 1 year that requires ongoing medical attention and/or limits activities of daily living^1^. Cardiovascular diseases (such as heart attacks and stroke), cancers, chronic respiratory diseases (such as chronic obstructive pulmonary disease and asthma), diabetes, and chronic renal diseases account for > 80% of all chronic disease-related deaths that occur before the age of 70 years^1,2^. Additionally, chronic diseases such as hypertension, dyslipidemia (high low-density lipoprotein cholesterol), and diabetes are major risk factors for cardiovascular diseases^1^. Regarding health-related role limitations, chronic arthritis is a leading cause of work disability in the United States and a common cause of chronic pain^1^. Chronic arthritis affects about one in four adults in the United States and is more prevalent among those diagnosed with diabetes and/or cardiovascular disease compared with adults without chronic diseases^3^. Osteoporosis is a major risk factor for all-cause mortality in adults aged ≥ 60 years^4^, and is associated with substantial health-related role limitations even in the absence of frailty fractures^5^. Globally, 30–60% of adults have multiple chronic conditions that can negatively affect health, function, and quality of life^6,7^. For instance, hypertension, diabetes, dyslipidemia, chronic pulmonary disease, chronic renal disease, chronic arthritis, and osteoporosis are frequently comorbid and associated with higher risks of all-cause mortality and health-related role limitations.

The likelihood of chronic diseases might depend on the combined effects of genetic, physiological, environmental, and behavioral factors^2^. For instance, lower socioeconomic status is associated with a higher risk of developing chronic diseases such as cardiovascular disease^8,9^. Moreover, in adults with multiple chronic diseases, lower educational achievement and monthly household income are associated with worse clinical status at follow-up assessments^10^. Accordingly, comorbid depressive symptoms and poor health-related behaviors, such as non-adherence to pharmacotherapy, reportedly mediated the relationship between lower socioeconomic status and uncontrolled blood pressure in middle-aged and older adults with hypertension^11^. To improve patient outcomes and potentially prevent chronic diseases, health-promoting behaviors such as maintaining a healthy diet, engaging in regular exercise and physical activity, getting enough sleep, quitting smoking, limiting alcohol intake, going for regular health screenings, making time for leisure activities, talking with friends about personal concerns and feelings, and participating in social activities within community- or faith-based organizations are crucial^12,13^. However, the vast majority of individuals newly diagnosed with a chronic disease do not adopt health-promoting behaviors and thus fail to achieve long-term improvements in health^14^.

### Health-related role limitations and health-promoting behaviors in chronic disease patients

Health-related role limitations are a major element of disease-related burden^15^. Chronic non-communicable diseases (i.e., diseases that are typically caused by unhealthy behaviors rather than being spread through infection) are an important cause of health-related disability and role limitation in developed countries^16^. The 12-item Short Form Survey (SF-12)^17^ can be used to measure limitations in physical health (i.e., general health, physical activities, and usual role activities [in association with physical health problems or bodily pain]) and mental health (i.e., vitality, emotional well-being, and usual role or social activities [in association with emotional problems]).

Health-related role limitations might be associated with decreased adherence to health-promoting behaviors in patients with chronic disease. Among adults with hypertension, dyslipidemia, and/or diabetes, those with fewer health-related role limitations in physical and mental health subdomains engaged in more health-promoting behaviors, specifically adequate fruit/vegetable intake and abstaining from smoking (which influenced mental health), and adequate physical activity (which influenced physical health, regardless of clinical status)^18^. Also, ego resilience had a stronger mediating effect on the association between emotional outlook and positive emotion at baseline versus after a health-promoting intervention aimed at increasing physical activity among adults in the workplace^19^. In contrast, in adults with hypertension, greater health-related role limitations in the mental health subdomain were associated with difficulty adhering to a healthy diet and engaging in regular exercise, as well as current smoking status^20^. Furthermore, the presence of psychological distress might decrease adherence to recommended preventive care, such as influenza vaccinations and annual dental check-ups^21^. Currently, the mechanisms underlying the association between health-related functional status and behavioral outcomes (i.e., health-promoting behaviors) are not well understood.

### Comorbid depressive symptoms and health-promoting behaviors in chronic disease patients

Chronic diseases such as hypertension^22^, diabetes mellitus^23^, pulmonary hypertension^24^, chronic kidney disease^25^, and rheumatoid arthritis^26^ are associated with increased rates of psychological distress, depressive symptoms, anxiety, and cognitive disturbance^27–29^. Furthermore, more severe comorbid depressive symptoms are associated with a higher number of chronic disease diagnosese^30^. Disease-related physical limitations and psychological distress, clinical deterioration, maladaptive health-related behaviors, and depressive symptoms are often mutually reinforcing^31,32^. Furthermore, comorbid depressive symptoms may affect health-related behaviors such as self-care behavior, physical activity, sleep time, eating habits, and compliance with treatment^31,33,34^, and were associated with adverse health and social outcomes in patients with chronic disease^27^. For example, depression negatively affects blood sugar control in diabetics because of lowered compliance to behaviors such as following a specific diet, taking medications on time, assessing metabolic parameters, and maintaining a consistent sleep cycle^35^. Furthermore, adults with comorbid depression are less likely to quit smoking after being diagnosed with acute coronary syndrome^36^. To date, few studies have examined the associations between health-promoting behaviors and depressive symptoms in patients with chronic disease^37–39^.

### Existential well-being and health-promoting behaviors in chronic disease patients

Existential well-being depends on an individual’s perspective on the meaning and purpose of life, satisfaction regarding their own life, and feelings regarding death and suffering^40^. The meaningful existence subdomain^40–42^ of the McGill quality of life questionnaire-revised (MQOL-R)^43,44^ measures existential well-being in terms of the meaning/purpose of life, progress/fulfillment of life goals, feeling of having control over one’s own life, and positive self-image. Existential well-being and health-promoting behaviors might have a bidirectional relationship. For instance, middle-aged adults with a greater sense of purpose in life are more likely to be physically active and less likely to experience sleep problems compared with those with less sense of purpose^45^. Furthermore, health-promoting behaviors, such as regular physical activity, at baseline were associated with a stronger purpose of life 4 years later^46^. Greater existential well-being has been linked with a lower rate of depression and better-preserved health. In patients with chronic renal disease undergoing peritoneal dialysis, fewer health-related role limitations in the physical and mental subdomains, as well as milder depressive symptoms, were correlated with a stronger sense of meaning and purpose in life^47^. Conversely, a diagnosis of stroke and/or depression at baseline appeared to contribute to a weaker sense of purpose 4 years later^46^. Existential well-being, i.e., a sense of control in life, is associated with emotional well-being (positive affect and optimism), less severe depressive symptoms, and health-promoting behaviors including physical activity, good sleep hygiene, and social involvement^48^. However, few studies have examined the item-level associations among health-related role limitations, depressive symptoms, facets of existential well-being, and diverse health-promoting behaviors in people with chronic diseases.

### Study aim and hypothesis

Physical and mental limitations (i.e., limitations in physical activities [due to health problems], role activities [due to physical health problems or bodily pain], vitality, emotional well-being, and usual or social activities [due to emotional problems])^49–51^, depressive symptoms^52,53^, existential well-being (meaning/purpose of life, progress/fulfillment of life goals, having control over one’s own life, and positive self-image)^40–42^, and socioeconomic status (educational attainment, employment, and monthly household income)^52–54^ are all associated with health-promoting behaviors (maintaining a healthy diet, engaging in regular exercise and physical activity, getting enough sleep, abstaining from smoking, limiting alcohol intake, going for regular health screenings, making time for leisure activities, talking with friends about concerns and feelings, and participating in social activities within community- or faith-based organizations)^12,13^ in adults with chronic diseases. Moreover, health-related role limitations in the mental and physical subdomains are associated with chronic disease and comorbid depressive symptoms in adults with chronic disease, where these relationships are mediated by health-promoting behaviors^51^. Adults with chronic diseases who had more severe depressive symptoms experienced greater enhancement of self-efficacy after an intervention targeting health-promoting behaviors^55^.

It can be difficult to capture the multiple mechanisms underlying health-promoting behaviors in adults with chronic disease, which include health status (health-related role limitations), cognitions (existential well-being), emotions (depressive symptoms), and behaviors (i.e., health-promoting behaviors)^56–58^, using correlation and regression analyses. Furthermore, it can be difficult to determine the variance in item-level responses among participants with similar summary scores^59,60^. To identify factors that should be targeted by interventions aimed at enhancing health-promoting behaviors in adults with chronic diseases, the relationships among assessment items must be examined^61,62^. To identify item-level associations among health-related role limitations, depressive symptoms, existential well-being, socioeconomic position, and health-promoting behaviors in adults with chronic disease, we used a network approach^38,59,63,64^. A mixed graphical model (MGM)^65^ comprised of continuous, categorical, and/or ordinal variables can be used to “regularize” partial correlations. In addition, directed acyclic graphs (DAGs)^63^ displaying probability distributions among variables, and the magnitude and direction of their relationships, can reveal conditional dependence. In the current study, we tested our hypothesis that there may be associations among health-related role limitations in physical and mental subdomains, vitality, emotional well-being, depressive symptoms, existential well-being, socioeconomic position, and health-promoting behaviors using a network approach. We also examined regularized partial correlations (MGMs) and “regularized directional independent associations” (DAGs) among assessment items. We expected to identify factors mediating the relationships of health-promoting behaviors with health-related role limitations, depressive symptoms, existential well-being, and socioeconomic status.

## Methods

### Participants and data collection

In this cross-sectional study, we recruited chronic disease patients who had been diagnosed at Seoul National University Hospital between October 2016 and February 2017. The inclusion criteria were as follows: aged ≥ 19 years, diagnosed with ≥ 1 chronic disease (hypertension^4,66^, hyperlipidemia^66^, diabetes^4,66^, chronic pulmonary disease^67,68^, chronic renal disease^69,70^, chronic arthritis^67,71^, or osteoporosis^4,5^), visited the outpatient clinic at Seoul National University Hospital between October 2016 and February 2017 for treatment of chronic diseases, fluent in Korean, and willing to participate in this study. A total of 535 adults regularly visited the outpatient clinic for treatment of chronic diseases and completed self-report questionnaires between October 5, 2016 and February 28, 2017. All participants received sufficient information about the study and provided written informed consent. The survey was anonymous and confidential. This study was approved by the clinical research ethics committee of Seoul National University Hospital College of Medicine (IRB number 1601-075-734). All procedures were performed in accordance with relevant guidelines and regulations.

### Measures

Demographic information (age, sex, marital status, residential area) and chronic disease diagnoses (hypertension, hyperlipidemia, diabetes mellitus, chronic pulmonary disease, chronic renal disease, chronic arthritis, and osteoporosis) were confirmed by a physician at the outpatient clinic. Information about socioeconomic status (educational attainment, monthly household income, employment status), health-related role limitations (assessed by the 12-item Short Form Survey [SF-12])^72^, depressive symptoms (assessed by the Patient Health Questionnaire-9 [PHQ-9]^73,74^, existential well-being (assessed by four items of the MQOL-R)^43,44^, and health-promoting behavior (assessed by the Healthy Habits Questionnaire [HHQ]^75^) was acquired; no clinical evaluations were conducted by psychiatrists.

#### Health-related role limitations: SF-12

**Figure 1:**
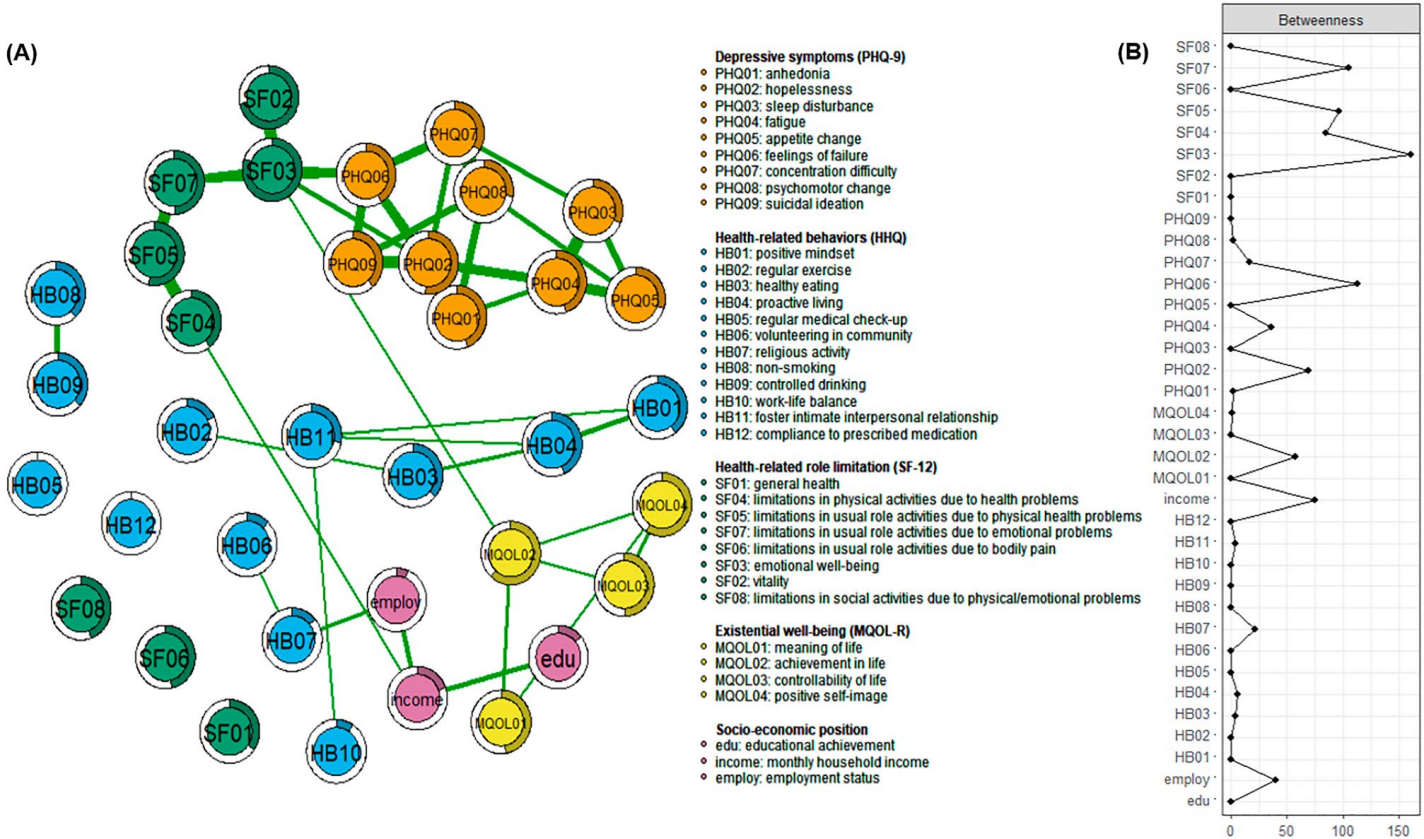
(**A**) Mixed graphical model comprised of health-related role limitation, depressive symptoms, existential well-being, and health behaviors in chronic disease. The “node predictability value” (variance in a given node’s value explained by the nodes with which it is connected) is indicated by the shadowed parts of rings surrounding each node. (**B**) Betweenness centrality values (the proportion of the shortest paths in the network containing a given node) of health-related role limitation, depressive symptoms, existential well-being, and health behaviors calculated from (**A**). The x-axis represents z-scores.

**Figure 2:**
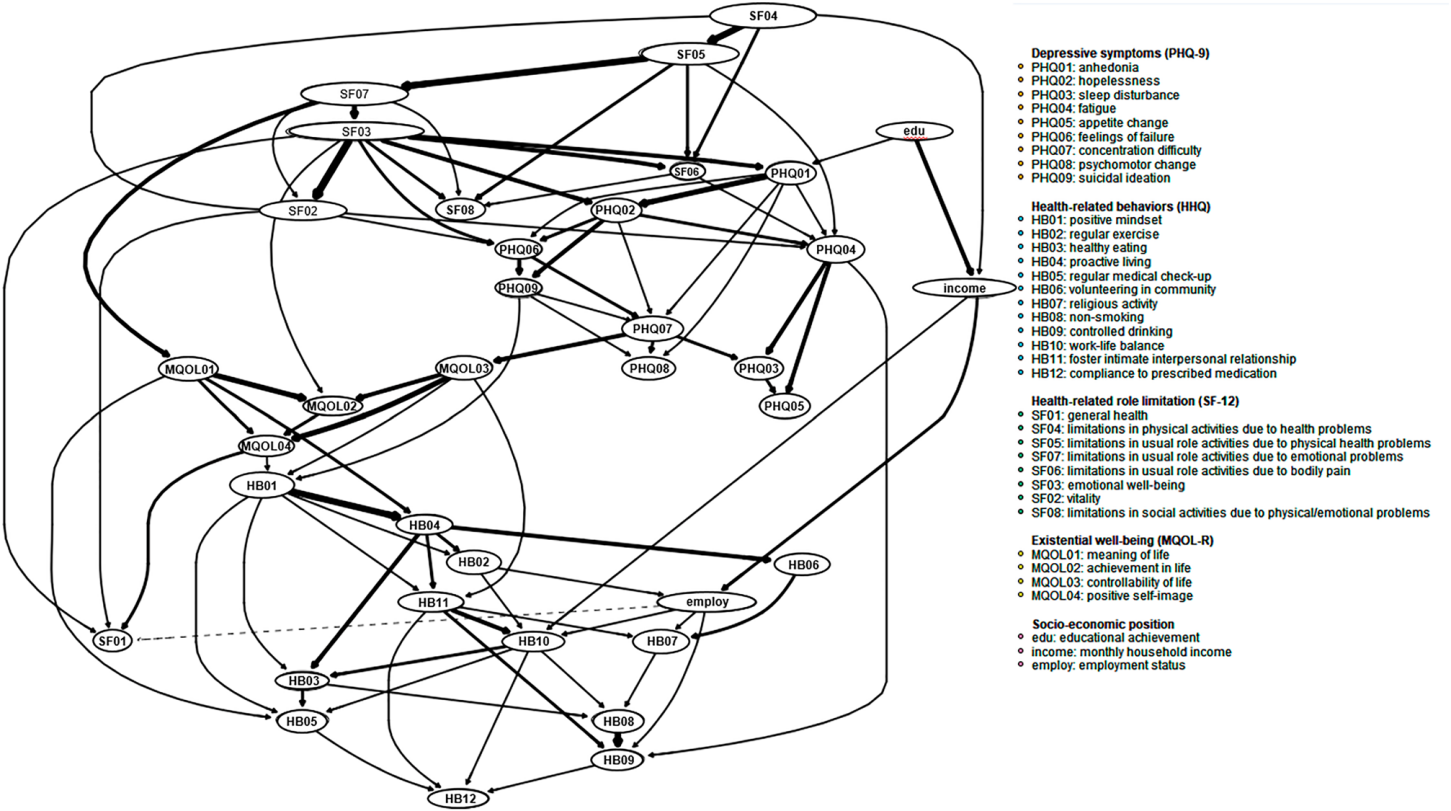
Directed acyclic graph of health-related role limitation, depressive symptoms, existential well-being, and health behaviors in chronic disease. The magnitude of association between two items is displayed by the thickness of an edge; the thicker the edge, the stronger the association between the two items connected.

The SF-12 was developed to measure health-related quality of life^17^. It consists of 12 items distributed among eight subdomains of physical and mental health^49^: including general health (item 1), vitality (item 10), emotional well-being (items 9 and 11), limitations in physical activities (moderate activities and climbing several stairs) due to health problems (items 2 and 3), limitations in usual role activities due to physical health problems (items 4 and 5), limitations in usual role activities due to bodily pain (item 8), limitations in usual role activities due to emotional problems (items 6 and 7), and limitations in social activities due to physical or emotional problems (item 12)^49,50^. The 5- and 3-point Likert scale scores for the items were averaged within each subdomain^49,50^ to be used as nodes in the MGM (Fig. 1) and DAG (Fig. 2).

#### Depressive symptoms: PHQ-9

The PHQ-9^73,74^ evaluates depressive symptoms including hopelessness, anhedonia, sleep disturbance, fatigue, changes in appetite, guilt, concentration difficulties, psychomotor agitation/retardation, and suicidal ideation via nine self-report items. The total scores on the PHQ-9 reflect the severity of depression, which is classified as no depression (score of 0–4), mild depressive symptoms (score of 5–9), moderate depressive symptoms (score of 10–19), or severe depressive symptoms (score of 20–27)^76^. The 4-point Likert scale scores (none, 3–4 days, 8–10 days, or 12–14 days) for the nine depressive symptoms were used as nodes in the MGM (Fig. 1) and DAG (Fig. 2).

#### Existential well-being: MQOL-R

The MQOL-R^43,44^ comprises 14 items and is used to measure the level of subjective well-being throughout the lifespan in terms of physical symptoms (3 items), psychological symptoms (4 items), social support (3 items), and meaningful existence (4 items)^40–42^. In the current study, the 10-point Likert scale scores for four items related to existential well-being (meaningfulness and purposefulness of life, progress/fulfillment of life goals, having control over one’s own life, and positive self-image as a person) were used as nodes in the MGM (Fig. 1) and DAG (Fig. 2).

#### Health-promoting behavior: HHQ

The HHQ^75^ examines the practice of 12 important health-promoting behaviors: positive mindset, regular exercise, balanced diet, proactive attitude, regular medical checkups, volunteering in the community, religious and existential activities, nonsmoking, controlled drinking, work-life balance, nurturing intimacy in social interactions, and consistently taking prescribed medications. Five-point Likert scale scores (no intention, intention of performing within the next six months, intention of performing within the next month, have been practicing for < 6 months, have been practicing for ≥ 6 months) were translated into binary data (have been practicing for any duration = 1, not practicing yet = 0) to be used as nodes in the MGM (Fig. 1) and DAG (Fig. 2).

### Statistical analysis

We used network analyses to elucidate the associations among the scores for the eight subdomains of the SF-12, nine PHQ-9 depressive symptoms (including suicidal ideation), four (MQOL-R) existential well-being items, 12 HHQ health-related habits, and three variables related to socioeconomic status (educational attainment, monthly household income, and employment status). “Undirected” and “directional conditional dependencies”^77^ among the variables were estimated using MGMs (undirected networks comprising nodes showing conditional dependencies or regularized partial correlations) and a DAG (a Bayesian network showing the conditional probability distributions and directional dependencies of nodes with parent nodes)^78,79^, respectively. In these networks, variables served as nodes (n = 36) and edges between nodes represented conditional dependence^59^.

#### MGM

This study included categorical, ordinal, and continuous data^80^, and we estimated “undirected conditional dependencies”^77^ between variables using MGMs with the R package *mgm*^65^. MGMs represent edge weights by generating node-wise regression coefficients^81^. To prevent potentially spurious associations, *mgm* employs the least absolute shrinkage and selection operator (LASSO) approach^82^. LASSO reduces all edge weights so that they approach zero and sets small weights to exactly zero^82^. To derive MGM networks, we optimized the edge weights during LASSO regularization (controlled by parameter λ) using a pairwise model (interaction order k = 2) and the extended Bayesian information criterion (tuning parameter γ = 0)^59^. Two variables were considered independent if they were not connected when conditioned on other variables^83^. The thickness of each edge in the network represents the strength of the association, with thicker edges representing stronger associations^84^. To identify the principal nodes within the MGM networks, we calculated the betweenness centrality (proportion of paths in the network that contain a given node)^85^; four items (nodes) within the top 12% were defined as hubs^38^. Similar to R^2^ regression coefficients, “node predictability values” were estimated, i.e., values reflecting how accurately a node could be predicted by the other nodes it shared an edge with^59,62,86^. Node predictability values were visualized using pie charts^62^. The MGM network structure was visualized using the Fruchterman − Reingold algorithm, which was performed using the R package *qgraph*^84^*.*

#### DAG

The DAG is a Bayesian approach for modeling networks with edges that are directed and noncircular, with the goal of discerning relationships among nodes based on cross-sectional data^63^. Using the R package *bnlearn*, we applied the ‘hill-climbing’ greedy search algorithm to our dataset^87^. The DAG added and removed edges (connecting variables or nodes), and reversed their direction, until the goodness of fit satisfied the BIC^88^. An iterative bootstrapping procedure with 10,000 iterations was used to determine whether an edge existed between two symptom nodes^88,89^. In the second step, we generated an averaged network by retaining edges that were consistently present in the 10,000 bootstrapped networks. The cut-off point for consistently present nodes was set using a statistical method with high sensitivity and specificity^88,90^. Finally, a BIC value was computed for each edge, with higher values (depicted by thicker edges) signifying greater importance within the network structure^88^.

## Results

### Sample characteristics

A total of 535 adults [mean ± SD age = 62.9 ± 11.9 years; males, n = 231; females, n = 304) participated in the current study. Table 1 provides the demographic, chronic disease diagnosis, socioeconomic status, health-related role limitation (SF-12 scores), depressive symptom (PHQ-9 scores), existential well-being (scores on four MQOL-R items), and health-promoting behavior (HHQ scores) data of the participants. The mean number of diagnosed chronic diseases per participant was 2.1 ± 0.9 and the most common diagnoses were hypertension (n = 284, 53.1%), hyperlipidemia (n = 279, 52.1%), and diabetes (n = 249, 46.5%). Regarding educational attainment, 230 (43.0%) participants had an education level of college or higher level, and another 183 (34.2%) were high school graduates. A total of 197 (36.8%) participants were employed (n = 96; 17.9%) or self-employed (n = 101, 18.9%), and another 338 (63.2%) were unemployed or retired. Only 196 (36.6%) of the participants had a monthly household income ≥ 3000 US dollars (third quintile of the monthly average income of households with one or more family members in the Republic of Korea during 2016 and 2017 [https://kosis.kr/]).

**Table 1:** Sample characteristics.

Item contents	Unit of response	Values	Abbreviations
Demographics			
Age [mean ± SD]	Years	62.9 ± 11.9	–
Sex [N(%)]	Male/female	231 (43.2%)/304 (56.8%)	–
Marital status [N(%)]	Married/(single, divorced, or widowed)	412 (77.0%)/123 (23.0%)	–
Residential area [N(%)]	Urban/rural	504 (94.2%)/31 (5.8%)	–
Chronic diseases			
Hypertension [N(%)]	Current diagnosis of chronic disease	284 (53.1%)	–
Hyperlipidemia [N(%)]		279 (52.1%)	–
Diabetes [N(%)]		249 (46.5%)	–
Chronic pulmonary disease [N(%)]		96 (17.9%)	–
Chronic renal disease [N(%)]		74 (13.8%)	–
Chronic arthritis [N(%)]		73 (13.6%)	–
Osteoporosis [N(%)]		67 (12.5%)	–
Socioeconomic position			
Educational attainment [N]	College or higher/high school/middle school/elementary school/none	230/183/67/52/3	Edu
Employment status [N(%)]	Employed or self-employed/unemployed or retired	197 (36.8%)/338 (63.2%)	Employ
Monthly household income [N]	< 800/800–1600/1600–2400/2400–3200/≥ 3200 US dollars	72/80/99/88/196	Income
Health-related role limitation: 12-item Short Form Survey (SF-12)			
General health status [mean ± SD]	Excellent(100)/very good(75)/good(50)/fair(25)/poor(0)	47.0 ± 21.3	SF01
Vitality [mean ± SD]	All the time(100)/most of the time(75)/some of the time(50)/a little of the time(25)/none of the time(0)	58.8 ± 31.7	SF02
Emotional well-being [mean ± SD]		67.4 ± 24.4	SF03
Limitations in social activities due to physical or emotional problems [mean ± SD]		82.7 ± 26.0	SF04
Limitations in usual role activities due to physical health problems [mean ± SD]	All the time(0)/most of the time(25)/some of the time(50)/a little of the time(75)/none of the time(100)	69.7 ± 29.6	SF05
Limitations in usual role activities due to emotional problems [mean ± SD]		77.1 ± 26.8	SF06
Limitations in usual role activities due to bodily pain [mean ± SD]	Not at all(100)/a little bit(75)/moderately(50)/quite a bit(25)/extremely(0)	75.1 ± 27.0	SF07
Limitations in physical activities due to health problems [mean ± SD]	Yes limited a lot(0)/yes limited a little(50)/no not limited at all(100)	66.8 ± 33.1	SF08
Depressive symptoms: patient health questionnaire-9 (PHQ-9)			
Anhedonia [mean ± SD]	Not at all(0)/2-6 days (1)/7–12 days (2)/nearly everyday (3) out of the last 2 weeks	0.6 ± 0.9	PHQ01
Hopelessness [mean ± SD]		0.5 ± 0.9	PHQ02
Sleep disturbance [mean ± SD]		0.7 ± 1.0	PHQ03
Fatigue [mean ± SD]		1.0 ± 1.0	PHQ04
Appetite change [mean ± SD]		0.5 ± 0.9	PHQ05
Feelings of failure [mean ± SD]		0.4 ± 0.8	PHQ06
Concentration difficulties [mean ± SD]		0.4 ± 0.7	PHQ07
Psychomotor change [mean ± SD]		0.2 ± 0.6	PHQ08
Suicidal ideation [mean ± SD]		0.2 ± 0.5	PHQ09
Existential well-being: McGill quality of life questionnaire-revised (MQOL-R)			
Meaning and purpose of personal existence [mean ± SD]	10-Point Likert scale: utterly meaningless and without purpose(0)-to–very purposeful and meaningful(10)	7.6 ± 2.2	MQOL01
Achieving life goals [mean ± SD]	10-Point Likert scale: made no progress whatsoever(0)-to-progressed to Complete fulfillment(10)	7.3 ± 2.2	MQOL02
Having control over one’s own life [mean ± SD]	10-Point Likert scale: no control over my life(0)-to-complete control over my life(10)	7.8 ± 2.1	MQOL03
Feeling good about oneself as a person [mean ± SD]	10-Point Likert scale: completely disagree(0)-to-completeky agree(10)	8.0 ± 2.0	MQOL04
Health-related behaviors: healthy habits questionnaire (HHQ)			
Positive mindset	No intension of practice(1)/intension of practice within 6 months(2)/intension of practice within 1 month(3)/have been practicing less than 6 months(4)/have been practicing more than 6 months(5)	4.4 ± 1.0	HB01
Regular exewrcise		4.1 ± 1.2	HB02
Healthy eating		4.3 ± 1.1	HB03
Proactive living		4.4 ± 1.0	HB04
Regular medical check-up		4.6 ± 0.9	HB05
Volunteering in community		3.5 ± 1.5	HB06
Religious activity		3.6 ± 1.8	HB07
Non-smoking		4.7 + 1.0	HB08
Controlled drinking		4.5 ± 1.1	HB09
Work-life balance		4.4 ± 1.0	HB10
Foster intimate interpersonal relationships		4.5 ± 0.9	HB11
Compliance to prescribed medication		4.8 ± 0.6	HB12

According to the SF-12 scores, the general health, physical functioning and role limitations due to physical health, and role limitation due to pain was fair, good, and very good, respectively. Vitality and emotional well-being were also good, and role limitations due to emotional problems and social functioning were very good. The severity of depressive symptoms, as evaluated using the PHQ-9, was mild (mean total PHQ-9 score = 4.4 ± 5.0). Regarding existential well-being, the median MQOL-R satisfaction (meaning/purpose of life, progress/fulfillment of life goals, feeling of having control over one’s own life, and self-image) score was 8 out of 10. Finally, the mean performance of health behavior (regardless of duration) score was 9.4 ± 2.8. The most commonly practiced health-promoting behaviors were consistently taking prescribed medications (n = 506, 94.6%) and abstaining from smoking (n = 485, 90.7%). The least frequently practiced health-promoting behaviors were volunteering in the community (n = 279, 52.1%) and religious and existential activities (n = 325, 60.7%).

### Network analyses: MGM and DAG

#### Overview

The MGM network for physical and mental quality of life (8 SF-12 domains), depressive symptoms (nine PHQ-9 items including suicidal ideation), existential well-being (four MQOL-R items), health-promoting behavior (12 HHQ items), and socioeconomic status (educational attainment, monthly household income, and employment status) is presented in Fig. 1A and Table 2. Although 636 edges were possible, only 42 (6.6%) were evident in the final MGM. According to the betweenness centrality data displayed in Fig. 1B, four items (nodes) within the top 12% of all 36 nodes comprising the MGM functioned as hubs in the MGM: emotional well-being (calm/peaceful and downhearted/sad; z-score = 3.25), limitations in usual role activities due to emotional problems (z-score = 1.92), physical health problems (z-score = 1.70), and feeling like a failure (z-score = 2.11). Furthermore, the degree to which each node could be predicted by the other nodes with which it shared an edge was estimated; the results are shown in Fig. 1A. In the DAG analysis (Fig. 2, Table 3), the strength of the association between two items is denoted by the thickness of the edge, as stated above^91^. Table 3 lists the edge weights (from strongest to weakest). The directions of the edge weights indicate the increase or decrease in a given score that would be expected if the arc were removed from the DAG^92^. If two items are strongly related, the edge weight will be negative and its absolute value will be large^91^.

**Table 2:** Weight adjacency matrix from the estimated mixed graphical model (MGM).

Items	PHQ01	PHQ02	PHQ03	PHQ04	PHQ05	PHQ06	PHQ07	PHQ08	PHQ09	HB01	HB02	HB03	HB04	HB05	HB06	HB07	HB08	HB09	HB10	HB11	HB12	SF01	SF04	SF05	SF07	SF06	SF03	SF02	SF08	MQOL01	MQOL02	MQOL03	MQOL04	edu	income	employ
PHQ01	0.00																																			
PHQ02	**0.43**	0.00																																		
PHQ03	0.00	0.00	0.00																																	
PHQ04	**0.08**	**0.20**	**0.30**	0.00																																
PHQ05	0.00	0.00	**0.14**	**0.23**	0.00																															
PHQ06	**0.09**	**0.20**	0.00	0.00	0.00	0.00																														
PHQ07	0.00	**0.13**	**0.10**	0.00	0.00	**0.22**	0.00																													
PHQ08	**0.17**	0.00	0.00	0.00	**0.08**	0.00	**0.39**	0.00																												
PHQ09	0.00	**0.27**	0.00	0.00	0.00	**0.22**	0.00	**0.15**	0.00																											
HB01	0.00	0.00	0.00	0.00	0.00	0.00	0.00	0.00	0.00	0.00																										
HB02	0.00	0.00	0.00	0.00	0.00	0.00	0.00	0.00	0.00	0.00	0.00																									
HB03	0.00	0.00	0.00	0.00	0.00	0.00	0.00	0.00	0.00	0.00	**0.04**	0.00																								
HB04	0.00	0.00	0.00	0.00	0.00	0.00	0.00	0.00	0.00	**0.09**	0.00	**0.05**	0.00																							
HB05	0.00	0.00	0.00	0.00	0.00	0.00	0.00	0.00	0.00	0.00	0.00	0.00	0.00	0.00																						
HB06	0.00	0.00	0.00	0.00	0.00	0.00	0.00	0.00	0.00	0.00	0.00	0.00	0.00	0.00	0.00																					
HB07	0.00	0.00	0.00	0.00	0.00	0.00	0.00	0.00	0.00	0.00	0.00	0.00	0.00	0.00	**0.03**	0.00																				
HB08	0.00	0.00	0.00	0.00	0.00	0.00	0.00	0.00	0.00	0.00	0.00	0.00	0.00	0.00	0.00	0.00	0.00																			
HB09	0.00	0.00	0.00	0.00	0.00	0.00	0.00	0.00	0.00	0.00	0.00	0.00	0.00	0.00	0.00	0.00	**0.12**	0.00																		
HB10	0.00	0.00	0.00	0.00	0.00	0.00	0.00	0.00	0.00	0.00	0.00	0.00	0.00	0.00	0.00	0.00	0.00	0.00	0.00																	
HB11	0.00	0.00	0.00	0.00	0.00	0.00	0.00	0.00	0.00	**0.02**	0.00	0.00	**0.02**	0.00	0.00	0.00	0.00	0.00	**0.03**	0.00																
HB12	0.00	0.00	0.00	0.00	0.00	0.00	0.00	0.00	0.00	0.00	0.00	0.00	0.00	0.00	0.00	0.00	0.00	0.00	0.00	0.00	0.00															
SF01	0.00	0.00	0.00	0.00	0.00	0.00	0.00	0.00	0.00	0.00	0.00	0.00	0.00	0.00	0.00	0.00	0.00	0.00	0.00	0.00	0.00	0.00														
SF04	0.00	0.00	0.00	0.00	0.00	0.00	0.00	0.00	0.00	0.00	0.00	0.00	0.00	0.00	0.00	0.00	0.00	0.00	0.00	0.00	0.00	0.00	0.00													
SF05	0.00	0.00	0.00	0.00	0.00	0.00	0.00	0.00	0.00	0.00	0.00	0.00	0.00	0.00	0.00	0.00	0.00	0.00	0.00	0.00	0.00	0.00	**0.30**	0.00												
SF07	0.00	0.00	0.00	0.00	0.00	**0.06**	0.00	0.00	0.00	0.00	0.00	0.00	0.00	0.00	0.00	0.00	0.00	0.00	0.00	0.00	0.00	0.00	0.00	**0.30**	0.00											
SF06	0.00	0.00	0.00	0.00	0.00	0.00	0.00	0.00	0.00	0.00	0.00	0.00	0.00	0.00	0.00	0.00	0.00	0.00	0.00	0.00	0.00	0.00	0.00	0.00	0.00	0.00										
SF03	0.00	**0.09**	0.00	0.00	0.00	**0.20**	0.00	0.00	0.00	0.00	0.00	0.00	0.00	0.00	0.00	0.00	0.00	0.00	0.00	0.00	0.00	0.00	0.00	0.00	**0.18**	0.00	0.00									
SF02	0.00	0.00	0.00	0.00	0.00	0.00	0.00	0.00	0.00	0.00	0.00	0.00	0.00	0.00	0.00	0.00	0.00	0.00	0.00	0.00	0.00	0.00	0.00	0.00	0.00	0.00	**0.30**	0.00								
SF08	0.00	0.00	0.00	0.00	0.00	0.00	0.00	0.00	0.00	0.00	0.00	0.00	0.00	0.00	0.00	0.00	0.00	0.00	0.00	0.00	0.00	0.00	0.00	0.00	0.00	0.00	0.00	0.00	0.00							
MQOL01	0.00	0.00	0.00	0.00	0.00	0.00	0.00	0.00	0.00	0.00	0.00	0.00	0.00	0.00	0.00	0.00	0.00	0.00	0.00	0.00	0.00	0.00	0.00	0.00	0.00	0.00	0.00	0.00	0.00	0.00						
MQOL02	0.00	0.00	0.00	0.00	0.00	0.00	0.00	0.00	0.00	0.00	0.00	0.00	0.00	0.00	0.00	0.00	0.00	0.00	0.00	0.00	0.00	0.00	0.00	0.00	0.00	0.00	**0.02**	0.00	0.00	**0.05**	0.00					
MQOL03	0.00	0.00	0.00	0.00	0.00	0.00	0.00	0.00	0.00	0.00	0.00	0.00	0.00	0.00	0.00	0.00	0.00	0.00	0.00	0.00	0.00	0.00	0.00	0.00	0.00	0.00	0.00	0.00	0.00	0.00	**0.03**	0.00				
MQOL04	0.00	0.00	0.00	0.00	0.00	0.00	0.00	0.00	0.00	0.00	0.00	0.00	0.00	0.00	0.00	0.00	0.00	0.00	0.00	0.00	0.00	0.00	0.00	0.00	0.00	0.00	0.00	0.00	0.00	**0.02**	**0.04**	**0.05**	0.00			
edu	0.00	0.00	0.00	0.00	0.00	0.00	0.00	0.00	0.00	0.00	0.00	0.00	0.00	0.00	0.00	0.00	0.00	0.00	0.00	0.00	0.00	0.00	0.00	0.00	0.00	0.00	0.00	0.00	0.00	0.00	0.00	0.00	0.00	0.00		
income	0.00	0.00	0.00	0.00	0.00	0.00	0.00	0.00	0.00	0.00	0.00	0.00	0.00	0.00	0.00	0.00	0.00	0.00	0.00	0.00	0.00	0.00	**0.03**	0.00	0.00	0.00	0.00	0.00	0.00	0.00	0.00	0.00	0.00	**0.08**	0.00	
employ	0.00	0.00	0.00	0.00	0.00	0.00	0.00	0.00	0.00	0.00	0.00	0.00	0.00	0.00	0.00	**0.05**	0.00	0.00	0.00	0.00	0.00	0.00	0.00	0.00	0.00	0.00	0.00	0.00	0.00	0.00	0.00	0.00	0.00	0.00	**0.09**	0.00

Edge weights indicate the strengths of conditional dependence relations between two different items connected by an undirected edge, which can be understood as partial correlations. With employment of least absolute shrinkage and selection operator (LASSO), all edge weights were shrunk toward zero and small edge weight values were set to exactly zero.

Significant values are in bold.

**Table 3:** Estimated edge weights in directed acyclic network.

Rank	Association direction		Edge weight	Rank	Association direction		Edge weight	Rank	Association direction		Edge weight
	From	To			From	To			From	To	
1	SF03	SF02	− 277.94	31	SF05	SF06	− 15.49	61	SF06	SF08	− 5.15
2	HB01	HB04	− 127.52	32	PHQ07	PHQ03	− 13.75	62	HB10	HB05	− 5.11
3	HB08	HB09	− 122.25	33	MQOL01	HB04	− 13.57	63	SF04	Income	− 4.77
4	SF05	SF07	− 114.54	34	SF04	SF06	− 13.52	64	HB07	HB08	− 4.59
5	SF04	SF05	− 104.36	35	SF03	SF08	− 13.07	65	SF02	PHQ04	− 4.31
6	SF07	SF03	− 103.35	36	SF05	SF08	− 12.33	66	PHQ01	PHQ06	− 4.18
7	PHQ01	PHQ02	− 97.15	37	MQOL04	SF01	− 12.03	67	SF04	SF02	− 3.85
8	MQOL01	MQOL02	− 67.44	38	PHQ06	PHQ07	− 11.60	68	SF07	SF02	− 3.80
9	MQOL03	MQOL04	− 65.48	39	HB04	HB11	− 11.30	69	PHQ09	PHQ07	− 3.74
10	PHQ04	PHQ03	− 63.42	40	HB06	HB07	− 11.28	70	SF02	PHQ06	− 3.73
11	SF03	PHQ01	− 63.13	41	HB10	HB03	− 10.75	71	SF07	SF08	− 3.56
12	HB11	HB10	− 55.96	42	HB03	HB05	− 10.63	72	SF06	PHQ04	− 3.44
13	MQOL03	MQOL02	− 54.86	43	HB04	HB02	− 10.54	73	PHQ04	HB09	− 3.15
14	SF07	MQOL01	− 43.47	44	HB11	HB09	− 9.76	74	HB01	HB02	− 2.78
15	Edu	Income	− 37.35	45	PHQ03	PHQ05	− 8.79	75	SF02	SF01	− 2.61
16	SF03	SF06	− 32.71	46	HB01	HB11	− 8.54	76	MQOL01	HB05	− 2.45
17	PHQ04	PHQ05	− 29.31	47	HB02	Employ	− 8.40	77	Income	HB10	− 2.41
18	HB04	HB03	− 28.20	48	Employ	HB07	− 7.94	78	Employ	HB09	− 2.40
19	HB04	HB06	− 28.05	49	HB11	HB07	− 7.80	79	HB10	HB12	− 2.14
20	SF03	PHQ02	− 25.98	50	HB02	HB10	− 7.10	80	PHQ02	PHQ07	− 1.97
21	PHQ07	MQOL03	− 25.35	51	SF03	MQOL02	− 7.04	81	Edu	PHQ01	− 1.93
22	PHQ02	PHQ09	− 24.23	52	PHQ01	PHQ08	− 6.80	82	MQOL04	HB01	− 1.74
23	PHQ06	PHQ09	− 22.32	53	HB05	HB12	− 6.79	83	MQOL03	HB01	− 1.71
24	PHQ02	PHQ06	− 20.69	54	HB11	HB12	− 6.67	84	PHQ01	PHQ07	− 1.60
25	PHQ02	PHQ04	− 20.23	55	HB01	HB05	− 6.47	85	Employ	HB10	− 1.58
26	PHQ07	PHQ08	− 19.85	56	HB10	HB08	− 6.47	86	SF03	SF01	− 1.39
27	MQOL02	MQOL04	− 18.72	57	HB09	HB12	− 6.22	87	HB03	HB08	− 1.21
28	SF03	PHQ06	− 18.37	58	PHQ09	HB01	− 6.00	88	PHQ01	PHQ04	− 0.93
29	MQOL01	MQOL04	− 17.35	59	MQOL03	HB11	− 5.55	89	HB01	HB03	− 0.92
30	Income	Employ	− 17.10	60	PHQ09	PHQ08	− 5.44	90	SF05	PHQ04	− 0.18

Directional weights with negative numbers and larger absolute values indicate stronger directional associations between two different items connected by directional edges in the directed acyclic network.

#### Perceived meaning, achievement of life goals, control of life and health-promoting behavior

Perceived meaning, achievement of life goals and control of one’s life were directly influenced by emotional problem-related limitations in usual role activities, emotional well-being (calm/peaceful and not sad), and concentration difficulties, respectively (Fig. 2, Table 3). There was a partial correlation between perceived achievement of life goals (existential well-being) and emotional well-being (calm/peaceful and not sad; r = 0.02), which contributed to the high predictive value (61.8%) for the achievement of life goals item (Fig. 1A, Table 2).

Positive mindset, as the hub node for health-promoting behavior in the DAG, was influenced by the joint probability distributions of suicidal ideation, perceived control of one’s life, and positive self-image. Positive mindset and perceived meaning of life (existential well-being) were both parent nodes of proactive living (health-promoting behavior). Furthermore, positive mindset, proactive living, and perceived controllability of one’s life were directly associated with the fostering of intimate interpersonal relationships (health-promoting behavior) (Fig. 2, Table 3).

The types of health-promoting behavior with the highest node predictability values in the MGM were proactive living (43.3%; via partial correlations with positive mindset [r = 0.09], balanced diet [r = 0.05], and nurturing intimacy in social interactions [r = 0.02]), positive mindset (40.0%; via partial correlations with proactive living [r = 0.09] and nurturing intimacy in social interactions [r = 0.02]), and nonsmoking (38.4%; via a partial correlation with controlled drinking [r = 0.12]) (Fig. 1A, Table 2). In the DAG, positive mindset had a direction influence on proactive living, nurturing intimacy in social interactions, regular medical checkups, regular exercise, and eating a balanced diet. Proactive living, which was influenced by the joint probability distributions of positive mindset and the meaning and purpose of life, also had direct associations with a balanced diet, volunteering in the community, nurturing intimacy in social interactions, and regular exercise (Fig. 2, Table 3).

#### Associations between health-related role limitations and depressive symptoms

Among the physical and mental health subdomains, node predictability values in the MGM were highest for emotional well-being (calm/peaceful and not sad; 80.3%) and vitality (71.4%), and lowest for general health (34.8%) and limitations in physical activities due to health problems (39.5%) (Fig. 1A, Table 2). In the DAG, limitations in physical activities due to health problems emerged was the most pivotal node, and had direct associations with limitations in usual role activities due to physical health problems and pain, monthly household income, and vitality. In turn, limitations in physical activities due to health problems had a direct influence on role limitations due to emotional problems and pain, limitations in social activities due to physical or emotional problems, and fatigue (Fig. 2, Table 3). Among the health-related role limitation items, the strongest partial correlations in the MGM were between limitations in usual role activities due to physical health problems and limitations in physical activities due to health problems; limitations in usual role activities due to physical health problems and those due to emotional problems; and emotional well-being (calm/peaceful and not sad) and vitality (r = 0.30 for all three relationships; Fig. 1A, Table 2).

Health-related role limitations and depressive symptoms were connected in the MGM through the regularized partial correlation between emotional well-being and feeling like a failure (r = 0.20; Fig. 1A, Table 2). In the DAG, feeling like a failure was affected by the joint probability distributions of emotional well-being, vitality, hopelessness, and anhedonia. The emergence of depressive symptoms in the DAG was related to anhedonia through the joint probability distributions of emotional well-being and educational attainment. Anhedonia had direct associations with other depressive symptoms (feelings of hopelessness), psychomotor retardation/agitation, concentration difficulties, fatigue, and feeling like a failure (Fig. 2, Table 3).

The predictability of depressive symptoms in the MGM included both low values, i.e., 27.7% (psychomotor agitation/retardation) and 29.9% (changed appetite and eating behaviors), and relatively high values, i.e., 45.0% (fatigue) and 52.0% (hopelessness). The strongest partial correlations among the depressive symptoms in the MGM were between hopelessness and anhedonia (r = 0.43), and psychomotor agitation/retardation and concentration difficulty (r = 0.39). Notably, the node predictability value of suicidal ideation was 38.5% due to its partial correlations with hopelessness (r = 0.27), feeling like a failure (r = 0.22), and psychomotor agitation/retardation (r = 0.15) (Fig. 1A, Table 2). Likewise, in the DAG, suicidal ideation was influenced by the joint probability distributions of feeling like a failure and hopelessness (Fig. 2, Table 3).

## Discussion

### The relationship between emotional well-being and the feeling of failure in individuals with chronic disease mediates the relationships of limitations in usual role activities due to emotional problems with concentration difficulties and suicidal ideation

In the current study, the most influential nodes in the MGM (indicated by the betweenness centrality values) in terms of the regularized partial correlations among items were emotional well-being (calm/peaceful and downhearted/sad), feeling like a failure, limitations in usual role activities due to emotional problems (low mood and anxiety), and limitations in physical activities due to health problems. Likewise, the DAG in the current study revealed direct associations of limitations in physical activities due to health problems with limitations in usual role activities due to physical health problems and/or bodily pain, monthly household income, and vitality. The prevalence of chronic disease is a significant predictor of depressive symptoms^93^. Physical symptoms such as pain, dyspnea, gait and balance problems, and frailty, in addition to low ability to accept illness and long illness duration, may contribute to reduced physical activity, impaired activities of daily living, limited role functioning in occupational and social settings, depressive symptoms, anxiety, and lower life satisfaction^94–99^. Medication for chronic disease combined with antidepressants^22^, behavioral inerventions^100^, cognitive-behavioral therapy^101^, and face-to-face social support^102^ could be helpful for improving the physical health status and decreasing the depressive symptoms of patients with chronic disease.

Our findings emphasize the importance of preserving the usual role activities and social functioning of patients with chronic disease. In both the MGM and DAG, regularized partial associations (MGM) and the joint probability distribution of “directional conditional dependencies” (DAG) between emotional well-being (calm/peaceful and downhearted/sad) and feeling like a failure mediated the relationships of limitations in usual role activities due to emotional problems with concentration difficulties and suicidal ideation. In the MGM, the predictability of suicidal ideation was 38.5% due to partial correlations with hopelessness, feeling like a failure, and psychomotor change. Previous studies have also suggested that factors potentially exacerbating suicidal ideation include hopelessness (pessimism about the future), concentration difficulties, low motivation levels, and distress related to serious medical illnesses^103–105^. In our DAG, suicidal ideation was dependent on the joint probability distributions of feeling like a failure and hopelessness. Additionally, suicidal ideation had direct associations with psychomotor changes, concentration difficulties, and a positive mindset. Combining health education^106^ (delivered using electronic mobile devices) with motivational interviewing^107^ could improve depressive symptoms and self-efficacy.

### Associations between the meaning/controllability of life and health-promoting behaviors

The current findings emphasize the importance of existential well-being to health-promoting behaviors. In the DAG, a positive mindset and proactive living mediated the relationships of existential well-being with health-promoting behaviors. Specifically, the health-promoting behavior of maintaining a positive mindset was influenced by the joint probability distributions of suicidal ideation, positive self-image, and feelings of control over one’s life. Perceived loss of control over one’s life was significantly associated with perceived unaffordability of long-term life projects^108^. In a life crisis, such as a new cancer diagnosis, people desire agency in daily life and interpersonal connections^109^. Therefore, improving the knowledge, skills, attitudes, and self-awareness necessary for good health behaviors could reduce anxiety and depressive symptoms in adults with chronic disease^110^.

In this study, both a positive mindset and strong sense of purpose in life had direct associations with other health-promoting behaviors of proactive living. Proactive living had direct associations with a balanced diet, regular exercise, volunteering in the community, and nurturing intimacy in social interactions. The importance of the meaning and purpose of life has been emphasized with respect to treatment adherence in people with newly diagnosed cancer^111^. Good health behaviors, such as adherence to prescribed medication, are difficult to achieve and maintain, especially in patients with chronic disease^112^. Conversely, for people faced with a life crisis, loss of meaning is strongly connected with suicidal ideation^104^. As the search for and presence of meaning in life are highly correlated in patients with chronic disease or pain, finding meaning in life must be a focus during the management of chronic disease patients^113^. People with physical illness and pain might find meaning and hope through personal activities and interpersonal relationships^111,113^. Regular assessments of interpersonal communication and social connections^109^, combined with cognitive-behavioral therapy^114^ and behavioral modification^115^, could enhance emotional well-being and life meaning^116^ in patients with chronic disease.

### Limitations

The current study had several limitations. First, as we used a cross-sectional design, establishing causality in the associations between the different variables was not possible. Second, we used self-report measures to examine health-promoting behavior. Real-time tracking of daily behaviors using wearable devices, such as smart watches, might provide more detailed and accurate measurements of health-promoting behavior. Third, we did not perform subgroup analyses or construct MGMs and DAGs for individual physical illnesses. However, the seven chronic diseases examined in this study (hypertension, diabetes, dyslipidemia, chronic pulmonary disease, chronic renal disease, chronic arthritis, and osteoporosis) are frequently comorbid, and 30–60% of adults have multiple chronic conditions^6,7^. Future studies with more participants are needed to compare network-level associations among different chronic diseases.

## Conclusions

We applied network analysis to identify factors that could be targeted by interventions to enhance health-promoting behaviors in adults with chronic diseases. The meaning and controllability of life of individuals with chronic disease mediated the associations of health-related limitations in usual role activities, physical activities, and depressive symptoms. Interventions targeting health-promoting behaviors, the meaning and controllability of life, limitations of usual role activities, and physical activities, as well as proactive screening and timely psychiatric consultations to address depressive symptoms (such as feelings of failure, concentration difficulties, and suicidal ideation) are needed.

## Data Availability

The datasets generated and analyzed during the current study are available from the corresponding author on reasonable request.
